# Reward Is Assessed in Three Dimensions That Correspond to the Semantic Differential

**DOI:** 10.1371/journal.pone.0055588

**Published:** 2013-02-13

**Authors:** John G. Fennell, Roland J. Baddeley

**Affiliations:** Experimental Psychology, University of Bristol, Bristol, United Kingdom; University of Sheffield, United Kingdom

## Abstract

If choices are to be made between alternatives like should I go for a walk or grab a coffee, a ‘common currency’ is needed to compare them. This quantity, often known as reward in psychology and utility in economics, is usually conceptualised as a single dimension. Here we propose that to make a comparison between different options it is important to know not only the average reward, but also both the risk and level of certainty (or control) associated with an option. Almost all objects can be the subject of choice, so if these dimensions are required in order to make a decision, they should be part of the meaning of those objects. We propose that this ubiquity is unique, so if we take an average over many concepts and domains these three dimensions (reward, risk, and uncertainty) should emerge as the three most important dimensions in the “meaning” of objects. We investigated this possibility by relating the three dimensions of reward to an old, robust and extensively studied factor analytic instrument known as the semantic differential. Across a very wide range of situations, concepts and cultures, factor analysis shows that 50% of the variance in rating scales is accounted for by just three dimensions, with these dimensions being Evaluation, Potency, and Activity [1]. Using a statistical analysis of internet blog entries and a betting experiment, we show that these three factors of the semantic differential are strongly correlated with the reward history associated with a given concept: Evaluation measures relative reward; Potency measures absolute risk; and Activity measures the uncertainty or lack of control associated with a concept. We argue that the 50% of meaning captured by the semantic differential is simply a summary of the reward history that allows decisions to be made between widely different options.

## Introduction

In a stationary world we should expect that options that have led to good results in the past will often lead to good results in the future. This intuition lies at the heart of reinforcement learning models such as Q-learning [2] and provides a simple method for making decisions. If we keep a running estimate of the positive and negative consequences associated with a given object of choice, then, when presented with a choice between two options, we ought to choose the one that has been associated with a higher positive reward. Reinforcement learning models like 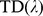 [3] offer a more formal account of such behaviour, where a temporally discounted estimate of reward is used (that is, more recent events contribute more strongly than those further away in time). Importantly, extensive recent research has indicated that a model of this basic type appears to be operating within the brain, where dopamine signals the reward prediction error, the critical parameter of such models [4]. Evidence for such reinforcement learning models has been provided by neurophysiology, fMRI, and behavioural experiments [5], [6]. The effectiveness of such a learning and decision making strategy, at least for small scale problems, has been confirmed by multiple computational experiments (for instance the method can be made to learn to play a very effective game of backgammon [7]).

These types of problems, maximising some sort of reward, have also been studied from the perspective of behavioural ecology. For example, McNamara and Houston [8] developed the idea of a common currency, in a similar way to that framed above, based on reproductive value and showed, using a dynamic programming approach, that many different costs can be used to explain a given behavioural sequence. Importantly, although reproductive value provides a common currency, value depends on context and is not fixed [9], and no guide to how options might actually be valued is provided; indeed reward values tend to be assumed (e.g. [10] p466). These ways of making decisions, by associating each decision object with a discounted history of reward and then choosing options that are likely to maximise that reward, is an effective method for computers to make decisions and has extensive neurophysiological and biological support. The approaches are, though, not without their problems; the most basic being that, in their simplest form, they are insensitive to the risks involved in achieving the reward.

As revealed rather graphically by the banking crisis of 2008 [11], simply maximising the probability of positive outcomes based on historical information is problematic and, potentially, disastrous. Not all options of equal average reward are equal in terms of risk and an agent that is insensitive to the risks associated with an option is liable to be out competed in the long run by one that is. Risk, we propose, comes in two related forms. Perhaps the simplest type of risk is the probability of a loss; “the quantifiable likelihood of loss or less-than-expected returns” ( [12] p1). At a minimum it would be advantageous for an agent to maintain not only an estimate of the average reward associated with an object of choice, but also the probability that it was associated with some bad outcome. In modern risk management this is often quantified using value at risk (VaR) and is akin to risk or ‘determinate uncertainty’ [13] as often used in the decision making literature. The second (related) form of risk, and historically the one concentrated on in financial contexts, is uncertainty, or more generally lack of knowledge and/or control (also referred to as ‘indeterminate uncertainty’ or ambiguity [14]).

That a richer representation of reward is used in decision making is not a novel suggestion. Evidence for representations of both uncertainty and risk has been provided based on research using imaging techniques [15], [16], theoretical treatments of neurotransmitters [17]–[20], and the basic reinforcement learning framework has been modified to allow the possibility of uncertainty [21], [22].

Here we propose to use a different sort of evidence to understand our representation of reward. Although most dimensions of a representation are specific to a given domain (has wings is relevant to describing birds, but not situations), since a decision can be about anything we know about, all of those things should have (under our suggestion) a three dimensional representation of reward. Therefore if we use some method to identify the dimensions underlying the “meaning” of things that we know about, and apply this to a very large range of things, because the reward based dimensions will be present for all of them (with the domain specific dimensions applicable only to particular sub-domains), the most important dimensions should turn out to be reward related. Identifying the underlying dimensions of the meaning of a wide range of things that we know about (henceforth concepts) is precisely what was performed, over 50 years ago in a very influential paper by Charles Osgood.

Osgood [1] developed a very simple method: The semantic differential. The semantic differential is probably the most successful empirical method devised for studying the nature of connotative (or affective) meaning. Establishing a semantic differential is procedurally simple: Concepts (e.g. objects, actions and settings) are presented to participants who are asked to rate them on perhaps as many as 50 scales (in the case of Osgood, May and Miron [23]; Landis et al [24] used 60 scales). Each scale is typically a seven point scale based on contrasting adjectives (e.g. clean vs dirty; fast vs slow etc.), with the central point being neutral. Factor analysis or principal components analysis is then used to determine the latent factors or dimensions underlying these ratings. This results in three robust findings: *a*) approximately 50% of the variance in the rating data can be captured by just three factors (usually named Evaluation, Potency and Activity or E, P and A); *b*) the most important of these, known as Evaluation, almost always corresponds to whether the concept is ‘good’ or ‘bad’; and *c*) the two other factors, Potency and Activity, each account for about the same amount of variance, with Potency capturing the extent the concept is ‘strong’ or ‘weak’, and Activity whether the concept is ‘calm’ or ‘chaotic’. Although the semantic differential technique and its basic findings, are over fifty years old, it has stood the test of time and has been found to be robust across domains [25], [26], languages and cultures [23], [27]. There are few principles that have received such between group verification or find application in so many areas; indeed, more than fifty years later (November 2012), a search of Google Scholar for the phrase “semantic differential”, restricted to “Since 2012” and not including patents or citations, revealed “About 2,300 results” that utilise the concept.

With the advent of the internet, approaches to the semantic differential have evolved, for example, an effective way of establishing a semantic differential is to use single scales for each of E, P and A with a set of adjectives defining either end of the scale [28]. Scales using discrete values have been replaced with sliders providing real numbers. In this way activity might be measured directly on a scale with adjectives such as quick, loud and active at one end, and slow, quiet and passive at the other.

Theoretically though, the semantic differential is less satisfactory [29], [30]. Besides the rather vague idea that the semantic differential measures connotative or affective meaning, it is still far from clear what is actually being measured and more importantly why such a robust, reproducible finding is found over so many domains and cultures. As is clear from our previous discussion, we propose that the three dimensions of the semantic differential represent a summary of the history of reward, specifically the average reward, the risk, and the level of uncertainty associated with a particular concept.

To test this hypothesis two things are needed: *a*) the measured location in the semantic differential space of a large number of concepts; together with *b*) a means to estimate the probability of positive and negatively rewarded situations associated with them. Obtaining the first of these is straightforward as there are semantic differential dictionaries publicly available [31]. However, it is more difficult to estimate the rewards and punishments associated with these concepts in everyday life.

At first sight, the problem of estimating the distribution of the probability of good or bad things happening across a wide range of contexts and concepts seems impossible. Ideally, but somewhat impractically, someone would be observed throughout their lifetime and for each of a large range of concepts, the number of times good and bad things were associated with them would be recorded (together with the number of times the context or concept occurred). This though is obviously impractical. Fortunately, a more pragmatic solution to the problem is provided by the recent phenomenon of the internet weblog or blog. Blogs are short descriptions of peoples' life experiences (good, bad and indifferent). They are also searchable. Blogs as whole are difficult to characterise as they cover such a wide and diverse range of areas. However, technorati.com produces a report annually, that is referred to as ‘State of the Blogosphere’, from a wide ranging survey of bloggers, and while the rigour of the data collection is not known, the latest report (based on N = 4114 responses in 2011) [32] characterises the blogosphere as follows: *a*) Hobbyists (61%) who receive no income from their blog; *b*) Professional part time (13%) who are paid as part of their full time job; *c*) Professional full time (5%) who are paid and consider it their full time job; *d*) Corporate (8%) who blog for an organization; and *e*) Entrepreneurs (13%) who blog for their own organization. In the report [32], the largest group, Hobbyists, say that they “blog for fun” and do not report any income. The report goes on to make the following comments: “Half of hobbyists prefer to express their ‘personal musings’ when blogging.” “60% indicate they spend less than three hours a week blogging.” “Because 72% blog to speak their minds, their main success metric is personal satisfaction (61%)”. References to randomly chosen ‘good’ [33] and ‘bad’ [34] blogs are provided below.

The rest of this paper firstly shows that two of the dimensions of the semantic differential (Evaluation and potency) are highly predictive of the history of good and bad events associated with a given concept. We then confirm that the relationship is causal by inducing changes to the semantic differential of an object by associating it artificially with rewards and punishments.

## Analysis One: The relationship between Reward and the Semantic Differential in Blog Entries

Two search engines were identified that provided extensive coverage of blogs, provided structured searching facilities, and did not prevent the use of automated search scripts. Technorati (technorati.com) and BlogScope (blogscope.com) are both free at the point of use and provide extensive coverage. Technorati is a commercial search engine funded by advertising that claimed (in June 2008) to index 112.8 million blogs, however, at the time of writing no up to date figure was available and, since it was used for the data gathering described here, has undergone extensive restructuring. At the time of writing Blogscope claimed to be monitoring over 52.50 million blogs with 1.3 billion posts and was being developed as part of a research project at the University of Toronto.

The semantic differential dictionary that we used consists of 1500 concepts grouped under four broad headings of Behaviours (actions that a person can perform), Identities (different kinds of individual), Settings (places or times where interactions might take place) and Modifiers (emotions, traits, and statuses), offering a broad selection for analysis. This dictionary, which was compiled during 2002/3 at Indiana University [31], was chosen because of it was publicly accessible and it was the largest single dictionary that could be found.

Matlab [35] scripts were written to submit each of the 1500 concepts from the semantic differential dataset to both of the blog search engines. Using this method we counted the number of posts that contained the concept, 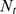; the number of posts containing the concept in combination with any of eleven unambiguously good words (good, amused, polite, relaxed, pleased, helpful, delighted, friendly, generous, honest, happy), 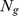, and the number of times the concept occurred in combination with ten unambiguously bad words (bad, suicidal, evil, abusive, cruel, depressed, miserable, rude, hurt, mean, unhappy), 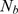. The good and bad word lists were chosen because they were the highest and lowest evaluated in the modifiers subgroup of the semantic differential dictionary that was used [31]. The distribution of the frequency of occurrence in spoken and written English of these words, taken from the British National corpus using their simple search (www.natcorp.ox.ac.uk), was not significantly different (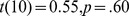).

From the original 1500 items in the semantic differential dictionary [31], two data sets were compiled each consisting of single word concepts that occurred at least once using each search engine. Technorati provided a data set consisting of 972 such concepts and Blogscope provided a data set consisting of 1071 concepts.

To simplify later analysis, we also calculated six measures derived from the positive and negative rewards associated with a concept. The first two measures simply measure the proportion of times the concept was associated with good and bad contexts, 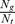, and 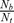 (which we call absolute positive and negative reward). The third measure quantifies the proportion of rewarded situations where this reward was positive, 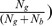 (relative reward). The fourth measure was a measure of frequency, 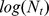. In addition, to investigate any interactions between the reward measures, the interaction between `negative and relative reward, 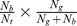, and ‘positive and relative reward’, 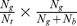, were also calculated. Using these as the independent measures, both correlational analysis, and stepwise regressions were performed against the three dimensions of the semantic differential (Evaluation, Potency and then Activity).

Of course, this technique of labelling blogs will produce noisy data. Posts will typically consist of many words, and a given concept may be very peripheral to the content of the blog in general. This method also ignores the complexities of English (e.g. “not bad” means good), and the method is completely blind to the magnitude of the reward (“kind of good” will be labelled the same as “very very good”). Fortunately, these effects should be random across concepts and balancing them is the absolutely vast amount of data available. Even if random effects are (very) large, by averaging over millions of blogs, any underlying relationship between reward and the location of the concept in the semantic differential space should still be detectable.

## Analysis One: Results and Discussion

We first analysed the best predictors of the evaluation dimension. Using both search engines, and both regression and correlation gave essentially the same result. Despite the previous provisos about the noisiness of our reward measures (and the fact that measured location in the semantic differential is not without noise), we obtained robust and strong relationships between our measures of reward and the evaluation dimension. The full results for the analyses are given in Tables 1 and 2 where the beta coefficient, b, standard error of the beta value, SEb, standardised coefficient, 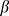, result of the *t*-test for the beta, *t*, multiple correlation coefficient, *R*, variance explained, 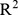, and the variance explained adjusted for the number of terms in the model, 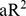, are given. This was dominated by the relative reward measure (technorati 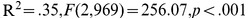; blogscope 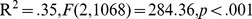). That this already strong relationship may in fact be stronger but hidden by some concepts being peripheral to the blogs is indicated by the fact that for the settings class (which is less likely to be peripheral to the content of a blog), the correlation coefficient reached 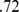 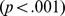. The results of the further multiple regressions, carried out for each sub group of concepts, are summarised in Tables 3 and 4 for the Technorati and Blogscope data respectively. The second most important predictor in all analyses (log probability of occurrence) also added weight to our interpretation of Evaluation as simply a measure of reward. The well documented “Mere exposure effect” [36]; an effect that informs much advertising, tells us that options will be preferred merely if they have been frequently observed (even if previous exposure is not associated with reinforcement).

**Table 1:** Stepwise multiple regressions (technorati).

DV	Step	IV	b	SEb	*β*	*t*	*r*	R^2^	aR^2^
E	1	(Constant)	−14.21	0.66					
		Relative reward	23.78	1.09	0.57	21.86	0.57	0.33	0.33
	2	(Constant)	−14.24	0.65					
		Relative reward	22.03	1.14	0.53	19.4			
		Exposure	0.12	0.02	0.13	4.83	0.59	0.35	0.34
P	1	(Constant)	2.08	0.13					
		Negative reward	−5.06	0.4	−0.37	−12.55	0.37	0.14	0.14
	2	(Constant)	0.45	0.26					
		Negative reward	−3.7	0.44	−0.27	−8.47			
		Exposure	0.13	0.02	0.23	7.1	0.43	0.18	0.18
	3	(Constant)	−0.1	0.3					
		Negative reward	−4.83	0.49	−0.36	−9.89			
		Exposure	0.13	0.02	0.23	7.08			
		Positive×Relative reward	3.32	0.67	0.16	4.96	0.45	0.2	0.2
A	1	(Constant)	1.67	0.14					
		Negative×Relative reward	−6.76	0.78	−0.27	−8.72	0.27	0.07	0.07
	2	(Constant)	0.85	0.27					
		Negative×Relative reward	−5.51	0.85	−0.22	−6.51			
		Exposure	0.06	0.02	0.12	3.55	0.29	0.09	0.08

Results of the stepwise multiple regressions for the Evaluation, Potency and Activity factors for the technorati data set. All 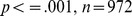.

**Table 2:** Stepwise multiple regressions (blogscope).

DV	Step	IV	b	SEb	*β*	*t*	*r*	R^2^	aR^2^
E	1	(Constant)	−9.06	0.42					
		Relative reward	15.88	0.72	0.56	22.05	0.56	0.31	0.31
	2	(Constant)	−10.11	0.43					
		Relative reward	14.47	0.73	0.51	19.93			
		Exposure	0.16	0.02	0.19	7.54	0.59	0.35	0.35
P	1	(Constant)	1.81	0.09					
		Negative reward	−3.62	0.25	−0.4	−14.36	0.4	0.16	0.16
	2	(Constant)	0.17	0.23					
		Negative reward	−2.9	0.26	−0.32	−11.04			
		Exposure	0.12	0.02	0.23	7.77	0.45	0.21	0.21
	3	(Constant)	−0.37	0.25					
		Negative reward	−3.4	0.28	−0.38	−12.35			
		Exposure	0.12	0.02	0.23	8.03			
		Positive×Relative reward	2.57	0.48	0.16	5.38	0.48	0.23	0.23
A	1	(Constant)	1.39	0.11					
		Negative×Relative reward	−4.81	0.55	−0.26	−8.81	0.26	0.07	0.07
	2	(Constant)	0.5	0.24					
		Negative×Relative reward	−4	0.58	−0.22	−6.92			
		Exposure	0.06	0.02	0.13	4.06	0.29	0.08	0.08

Results of the stepwise multiple regressions for the Evaluation, Potency and Activity factors for the blogscope data set. All 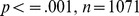.

**Table 3:** The amount of variance accounted for (R^2^) in the Technorati data from multiple regression for each factor for each subgroup of concepts using the predictors identified in the stepwise multiple regression.

Group	Dim	*r*	R^2^	*F*	*p*
	E	.46	.21	33.54	<.001
Behaviour *n* = 261	P	.36	.13	12.90	<.001
	A	.14	.02	2.78	= .063
	E	.63	.40	118.12	<.001
Identity *n* = 360	P	.40	.16	23.29	<.001
	A	.32	.10	19.71	<.001
	E	.66	.44	102.28	<.001
Modifier *n* = 262	P	.54	.29	34.32	<.001
	A	.28	.08	10.60	<.001
	E	.72	.52	46.13	<.001
Setting *n* = 89	P	.48	.23	8.22	<.001
	A	.10	.01	0.47	*n/s*

**Table 4:** The amount of variance accounted for (R^2^) in the Blogscope data from multiple regression for each factor for each subgroup of concepts using the predictors identified in the stepwise multiple regression.

Group	Dim	*r*	R^2^	*F*	*p*
	E	.47	.22	42.10	<.001
Behaviour *n* = 305	P	.36	.13	15.04	<.001
	A	.14	.02	2.86	= .05
	E	.61	.37	116.02	<.001
Identity *n* = 392	P	.40	.16	23.77	<.001
	A	.30	.09	18.62	<.001
	E	.71	.50	130.36	<.001
Modifier *n* = 263	P	.58	.34	45.20	<.001
	A	.30	.09	13.00	<.001
	E	.66	.44	42.76	<.001
Setting *n* = 111	P	.45	.20	8.82	<.001
	A	.10	.01	0.47	*n/s*

Perhaps as interesting as the sizeable correlations is the apparent independence of Evaluation from absolute (positive or negative) reward; evaluation does not simply measure the probability of good events being associated with a concept, only the proportion of rewarded events where the reward was positive.

In contrast, potency was most strongly correlated with absolute negative reward probability (technorati 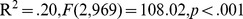; blogscope 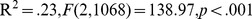). Again the next best predictor was our frequency measure, frequent concepts tend not only to be better evaluated but also labelled as more potent.

**Figure 1:**
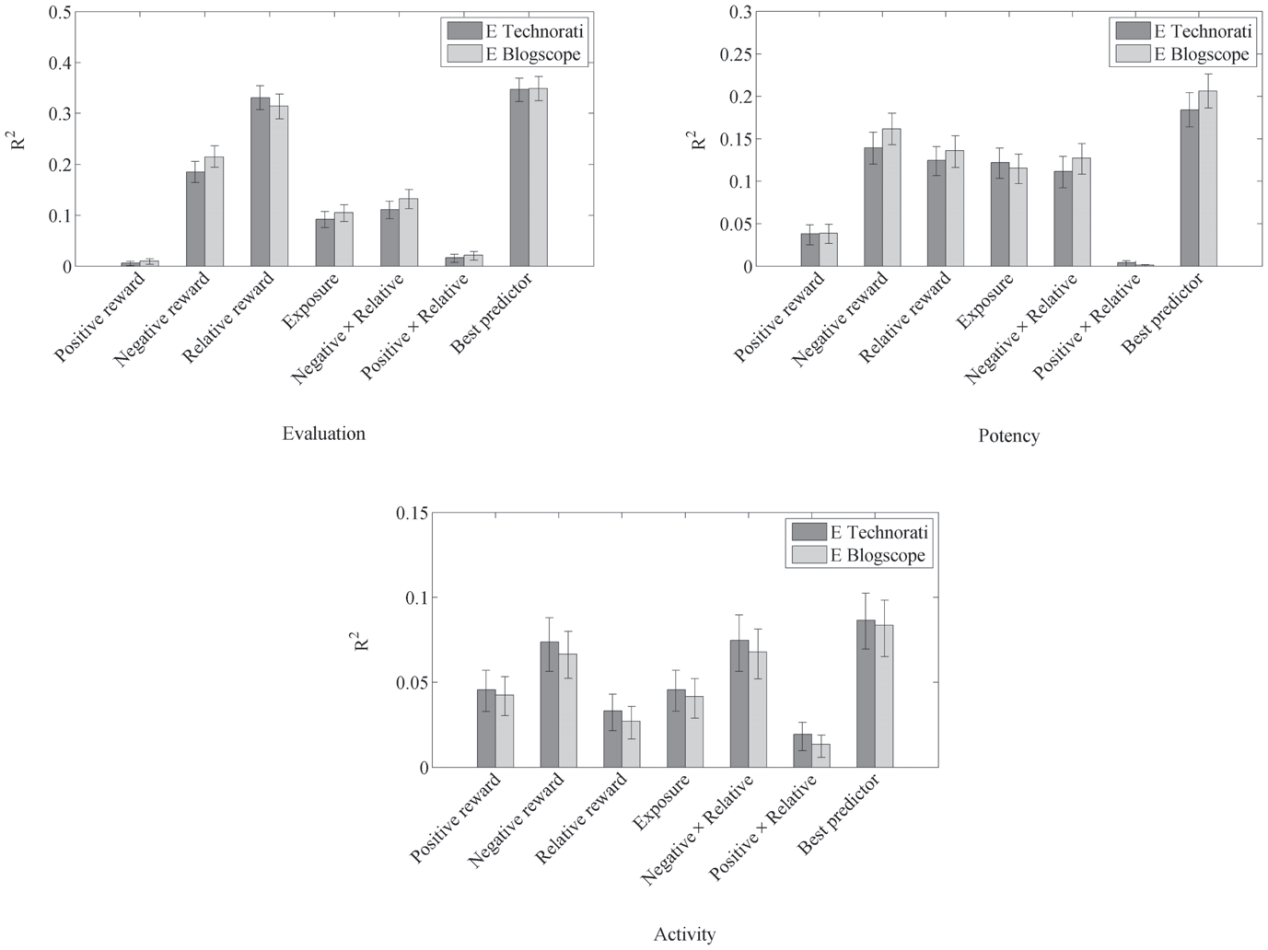
Left panel: The amount of variance accounted for 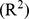 between Evaluation and each of the derived measures of reward. This clearly shows that relative reward is the best single predictor of evaluation. Dark bars represent the results using the technorati search engine, and light bars show the results for blogscope. The bars labelled ‘best predictor’ represent the adjusted 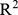 for the best fitting multiple regression model. Right panel: The amount of variance accounted for 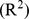 between Potency and each of the derived measures of reward. This shows that the best predictor of Potency is the probability of bad events (risk), though a number of other predictors are of reasonable size on their own. Bottom panel: The amount of variance accounted for 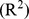 between activity and each of the derived measures of reward. The main observation is that though many of the measures of reward are significantly correlated with activity, the absolute level of correlation is small.

Lastly the best predictor for Activity was the ‘negative×relative’ reward interaction term, 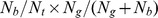. Though this relationship was highly significant, the level of correlation was moderate at best (technorati 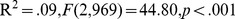; blogscope 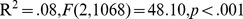). Figure 1 illustrates the amount of variance accounted for E, P and A for the derived measures of reward.

**Figure 2:**
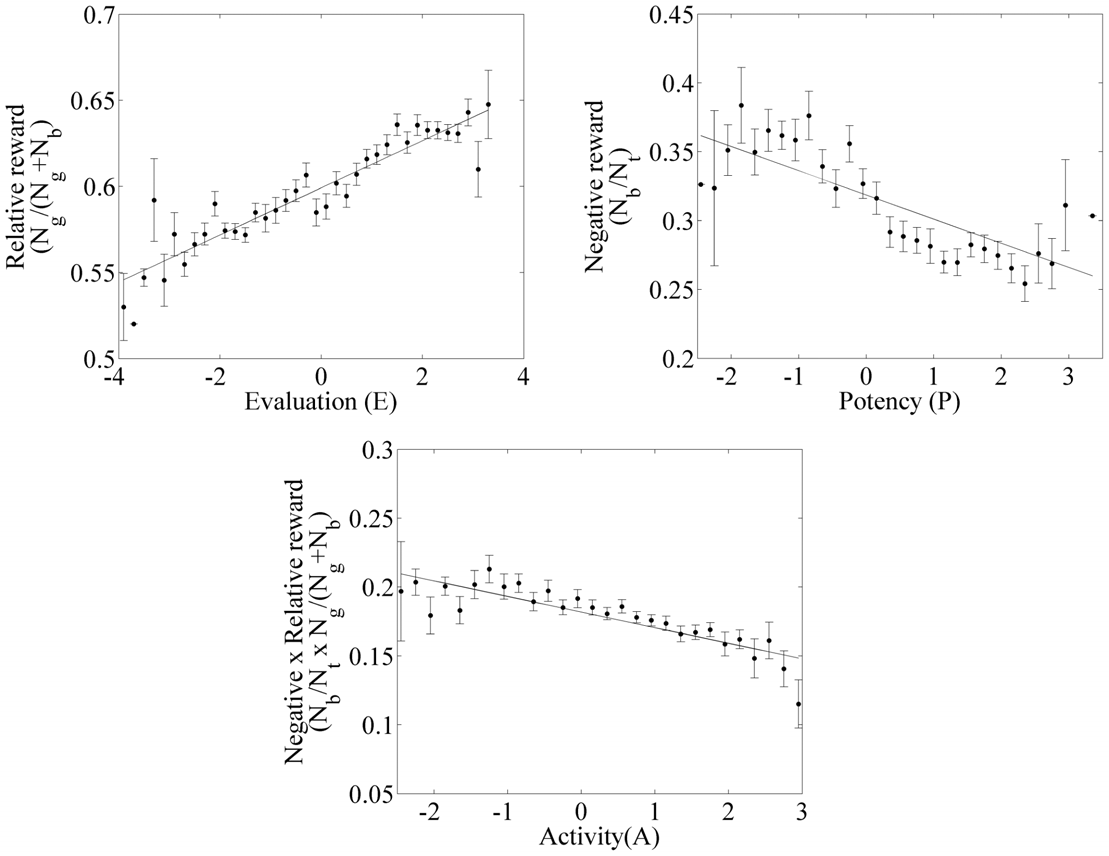
The relationships between the three semantic differential factors and their respective experienced rewards. To help understand the functional form of this relationship, the mean of the best measure of reward is calculated with bins of size 0.2. Error bars correspond to standard errors. As can be seen, though there are deviations, in each case the relationships are very close to linear.

Given the high level of noise associated with our method of evaluating reward and the inherent noisiness of the semantic differential, the correlations could, perhaps, be considered surprisingly strong. In order to gain further insight into the relationship between the semantic differential factors and the measures of reward, and to average over some of the noise associated with our methods of labelling blog entries, each of the three factors of the semantic differential was binned into bins of 0.2 and the average of the relevant reward measure calculated for all blogs within that range. This allows us to identify the underlying relationship, and in particular to see if the relationships are linear. Doing this showed that, once the effect of evaluation noise has been reduced, *a*) the relationships between the reward measures and the three dimensions of the semantic differential are very strong (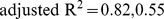 and 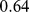 respectively, all 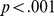); and *b*) the relationships are very close to linear. Not only is the semantic differential a measure of reward, but, as illustrated in Figure 2, it is a linear measure.

**Figure 3:**
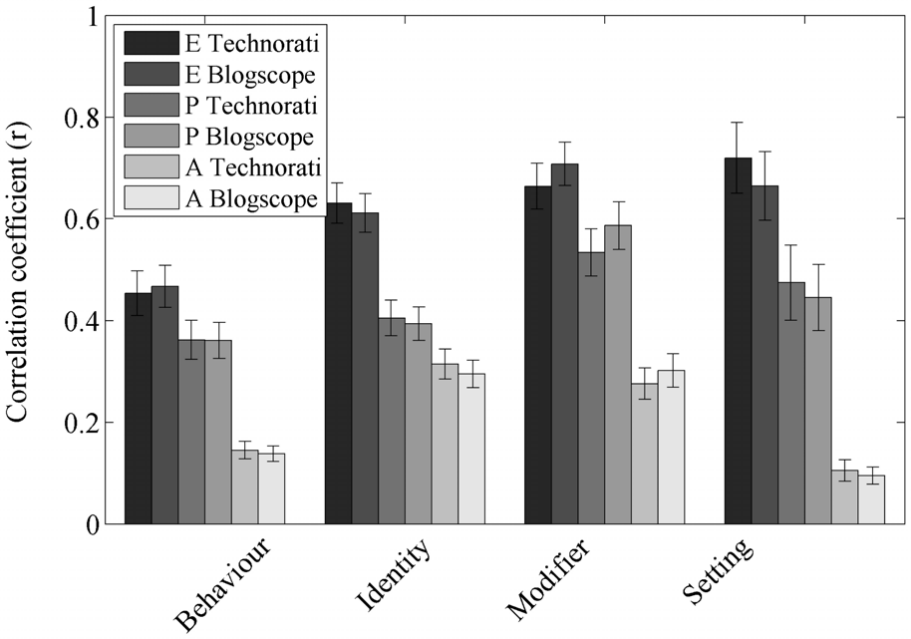
How the predictability varies between the semantic categories of the objects analysed. Shown is the correlation coefficient of the best fitting multiple regression model and Evaluation (darkest bar), Potency (lighter) and Activity (lightest). The technorati data set is shown as the left and blogscope as the right of the pair of bars. It can be seen that 1) the correlations are really very high, and 2) they are significantly higher for modifiers and settings, probably because these are usually more central to the blogs they feature in.

The concepts we analysed were subdivided into four categories: Behaviour (actions that one person can perform on another person), Identities (different kinds of individual), Settings (places or times where social interactions might take place) and Modifiers (emotions, traits, and statuses that might characterise people) [31]. Figure 3 illustrates that the predictability of the three dimensions of the semantic differential varies considerably between these categories. As already stated for instance, concepts within the settings category reached a correlation coefficient of .72 (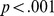) for predicting Evaluation. Again we interpret this in terms of the noise induced by our labelling process. Settings will more often be central to a blog, and hence the evaluation of the blog will be more aligned with that of the concept. Again we propose that this is evidence that the underlying relationship are even stronger than the (already large) ones measured here.

To summarise, we are proposing that two representations from very different disciplines, the semantic differential and reward, are in fact measures of the same thing. We have shown that three reward dimensions can be estimated from descriptions of good and bad experiences and, in addition, at least two dimensions of the semantic differential for a concept are strongly related to the distributions of rewarded events associated with them, with a third more moderately related to an interaction. Almost all objects, actions and contexts need at some time to be compared with others (do I attend to this object or that, do I perform this action or another…). To do this we need to have an estimate of the reward associated with each alternative. We propose that the semantic differential is this representation and “connotative meaning” is, to first approximation, a summary of reward history.

However, correlation does not imply causality. In order to establish this, we conducted a further experiment where arbitrary coloured shapes were associated with different distributions of positive, negative and neutral events. According to our hypothesis, simply associating these shapes with different reward histories should be sufficient to change the evaluation of them in terms of the semantic differential.

## Analysis 2: Changing Semantic Differential Evaluation by Changing Reward History

**Figure 4:**
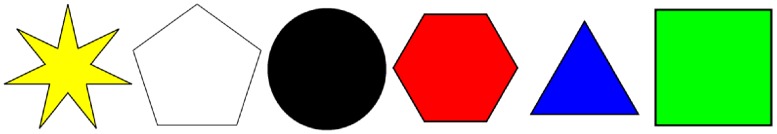
The shapes used for the rating and betting experiments.

This experiment manipulated the reward history associated with a number of coloured shapes (see Figure 4), with two conditions: pre-reward, and post-reward rating. The dependent measure was the difference in the semantic differential rating of a shape before and after the shape was associated with positive and negative rewards. A between subjects design was used to minimise the effects of initial rating on any post reward judgements. Two semantic differential ratings of the objects were conducted (by different participants), both before and after exposure to rewards being associated with them (with these rewards being the result of “bets” repeatedly being placed on the objects). Both ratings were performed using a web based program and the reward histories were manipulated by changing the probability of gains, losses and no-results being associated with the shapes.

Ethics statement: volunteers gave their informed written consent in accordance with the Declaration of Helsinki, and the experiments were approved by the Ethical Committee of the School of Experimental Psychology, University of Bristol.

### Participants

Forty participants performed the initial ratings, and sixty five rated the shapes after these were associated with different reward histories. All participants that volunteered were recruited by word of mouth and personal request and received no compensation for taking part. The mean age of the initial raters was 27.74 years, *SD* = 8.45 and 26.4 years, *SD* = 8.5 for the subjects providing the post reward exposure ratings. All participants reported normal or corrected to normal vision and all participants provided their informed consent prior to commencement of the experiment.

### Materials

The experiment was accessible via the internet, with both conditions implemented using a PHP script. Participants could choose to undertake it at a time and location that was convenient to them. The pre-reward condition simply consisted of the rating the shapes, and the post-reward condition started with an initial betting phase, followed by a rating phase. The shapes that participants were asked to rate are shown in Figure 4.

### Design

**Figure 5:**
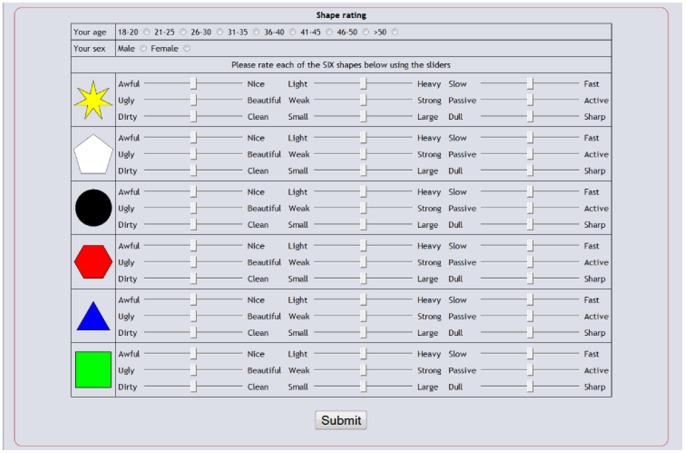
Browser based rating page used in the pre and post-reward conditions.

Both pre and post-reward evaluations used a repeated measures design. The rating page was a simple, one screen implementation that collected brief demographic information and presented all of the shapes to be rated. For each shape nine sliders were presented to the participant with each slider presented between two adjectives of opposite meaning. A button was included at the bottom of the screen in order to allow results to be submitted. The rating page is shown in Figure 5.

For the betting phase, the shapes were presented in distinct horizontal positions across the screen. The presentation locations were randomised for each participant but maintained for an experimental session, as it was reasoned that the position of the shape might influence betting amounts or ratings. The order that the shapes were presented in was randomised. For each random presentation of a shape whether a win or loss would happen was calculated using the distributions shown in Table 5.

**Table 5:** Means for distributions governing the occurrence of a win, loss or indeifferent outcome.

Shape	Probability of win	Probability of loss	Probability of indifferent	Distribution type
Star	0.0850	0.7650	0.1500	Bad
Pentagon	0.1625	0.4875	0.3500	Bad
Circle	0.2750	0.2750	0.4500	Indifferent
Hexagon	0.2750	0.2750	0.4500	Indifferent
Triangle	0.4875	0.1625	0.3500	Good
Square	0.7650	0.0850	0.1500	Good

Each distribution has a standard deviation of 0.05.

For each experimental trial, first, whether an event would happen was calculated and then, if an event was to happen, whether that event would be a reward or a punishment was calculated. Distributions were created so that shapes with the highest probabilities of an event happening attracted the highest probability of reward or punishment.

### Procedure - Ratings

The ratings phase, which was common to both the pre and post reward conditions, used nine scales. The scales were implemented as slider bars, which were used to rate each shape (see Table 6). The scales were the three identified as the most heavily loaded for each of Evaluation, Potency and Activity in a factor analysis carried out by Osgood ([37], p336) and were typical of the reliable scales used in other semantic differential studies. Each slider was initialised to the middle of the scale and the extremes were labelled 0 and 100. Participants moved the slider towards one extreme or the other, as indicated by the adjective, in order to record their rating.

**Table 6:** The nine scales used for rating the shapes.

Evaluation	Potency	Activity
Awful–Nice	Light–Heavy	Slow–Fast
Ugly–Beautiful	Weak–Strong	Passive–Active
Dirty–Clean	Small–Large	Dull–Sharp

### Procedure - Pre-reward

On entering the URL for the experiment, participants were presented with brief instructions and were asked to rate every shape by using every slider bar. Once all of the ratings were completed, participants were asked to press the submit button at the bottom of the page and were then presented with a thank you page, from which they could navigate away from the experiment.

### Procedure - Post-reward

**Figure 6:**
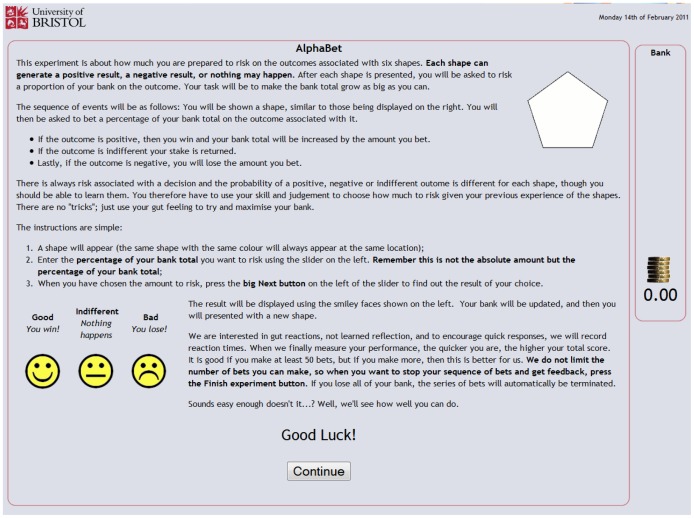
Instructions page.

**Figure 7:**
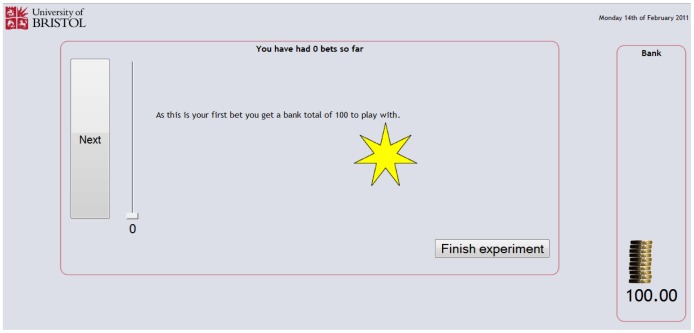
Initial betting page.

For the post-reward condition, once the URL for the experiment was entered and consent provided, participants were presented with a page of instructions (Figure 6). The instructions page explained that the experiment was concerned with making choices when some information was unknown, but could be learned. It was further explained that choices would be associated with six shapes and that each shape could generate a win, a loss, or nothing may happen. It was emphasised that there were no right or wrong answers and that what was of interest was ‘gut reactions’, in order to encourage quick responses. Along with the first randomly presented shape, participants were informed that they had been given an initial banked total of 100 (see Figure 7). For each presentation of a shape the participant was asked to risk a proportion of the banked total on the outcome, with the aim of building as large a banked total as possible.

**Figure 8:**
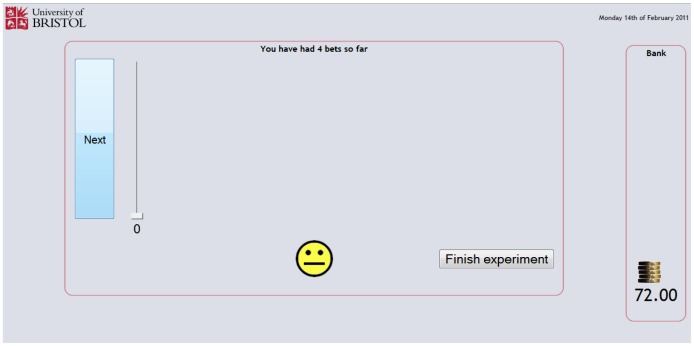
Feedback page.

**Figure 9:**
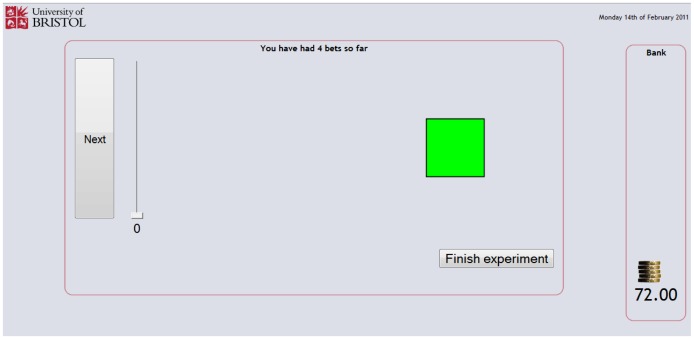
Betting page.

The web pages for the betting component of the experiment are shown in Figures 6, 7, 8 and 9, and provided a means for participants to place bets on the shapes shown in Figure 4 and receive feedback on those bets. If a win (good outcome) occurred with the shape the participant was returned the stake and won the equivalent amount, increasing the banked total; if a loss (bad outcome) occurred with the shape the participant lost the amount bet from the banked total; and if the event was indifferent the participant was returned the stake and the banked total was unaltered. After specifying the amount to bet on the currently displayed shape and pressing the next button, the participant was given feedback about the outcome of the event involving the shape using smiley faces displayed for a short duration (1000 ms). For a win a conventional smiley face was displayed (with the mouth turned up), for a loss a sad ‘smiley’ face was displayed (with the mouth turned down) and for an indifferent outcome a neutral ‘smiley’ face was displayed (with the mouth displayed as a horizontal straight line). An example of this feedback is shown in Figure 8. After the feedback had been provided the experiment continued with the next randomly generated shape (Figure 9).

The experiment was self paced with no limit to the number of bets that participants were able to make and the experiment could be terminated at any time by pressing the ‘Finish experiment’ button; participants were, however, asked to make at least fifty bets as this quantity was thought sufficient to ensure that they experienced the full range of the experimental shapes and their distributions. It was possible for participants to go ‘bust’ if a stake of 100% of the banked total was placed on what turned out to be a loss, in which case the ratings screen was displayed automatically, as if the ‘Finish experiment’ button had been pressed.

On finishing the experiment (or going bust), participants were presented with a rating page requesting brief demographic information and presenting all of the shapes to be rated based on the same rating scales as the initial ratings experiment (Figure 5 and Table 6). Once all of the ratings were completed, participants were asked to press the submit button at the bottom of the page and were then presented with a thank you page, from which they could navigate away from the experiment.

## Analysis 2: Results

The rating data for one participant were removed due to incomplete use of all of the rating scales, leaving ratings for thirty nine participants for the analysis.

### Factor Analysis

The data collected from participants for each of the rating scales were first checked for ‘factorability’ using the Kaiser-Meyer-Olkin measure of sampling adequacy (.68) and with Bartlett's test of sphericity (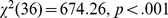). On the basis of these checks, further analysis was carried out on the data. Three principal components with eigenvalues greater than 1 were revealed, accounting for 67.36% of the variance in the ratings. Table 7 shows loadings of the component matrix following a varimax rotation.

**Table 7:** Rotated Component Matrix.

Scale	Factor 1	Factor 2	Factor 3
Awful–Nice		0.867	
Ugly–Beautiful		0.878	
Dirty–Clean		0.518	
Light–Heavy			0.736
Weak–Strong			0.802
Small–Large			0.579
Slow–Fast	0.867		
Passive–Active	0.856		
Dull–Sharp	0.743		

Factor loadings greater than .5 are shown from principal component analysis of the shape ratings, with varimax rotation which converged in five iterations, that were provided for the semantic differential scales in the ratings only experiment.

Though the factors were in a slightly different order from previous semantic differentials, three factors, consistent with previous findings, were revealed by the analysis, each containing three variables. The first related to rating scales indicating Activity, the second to rating scales indicating Evaluation and the third to rating scales indicating Potency. We note that the star shape may have unusually high initial activity and the black circle may have high initial levels of potency [38], however, because we are interested in the pre and post-betting differences in ratings, their effect is largely removed. The factor score coefficients calculated for each scale are shown in Table 8.

**Table 8:** Factor score coefficients resulting from principal component analysis of the shape ratings provided for the semantic differential scales in the ratings only experiment.

Scale	Factor 1	Factor 2	Factor 3
Awful–Nice	0.004	0.399	0.076
Ugly–Beautiful	−0.067	0.59	−0.007
Dirty–Clean	−0.046	0.05	−0.113
Light–Heavy	−0.023	−0.014	0.451
Weak–Strong	0.057	0.029	0.352
Small–Large	−0.074	0.012	0.225
Slow–Fast	0.371	−0.074	0.079
Passive–Active	0.425	−0.052	0.127
Dull–Sharp	0.219	−0.019	−0.052

If the connotative meaning of these shapes was to be altered, then presumably the results found here are the initial values that need to be changed. To test if the semantic differential can be changed in the directions suggested by the previous analysis, these initial ratings were compared to those found after the shapes were associated with different reward histories.

### Betting

Thirty eight participants were excluded from the post-betting analysis. Eighteen out of the thirty eight excluded participants chose not to complete the fifty bets requested, while eight were unable to complete enough bets because they went bankrupt and taken automatically to the rating page; the remainder provided fewer than fifty percent of the semantic differential ratings that were requested at the end of the experiment. More than 57% of the excluded participants had been presented with every shape fewer than five times, seven participants having had zero or only one presentation of at least one of the shapes. For the remaining participants, the mean number of trials undertaken was *M* = 108.56, *SD* = 46 with each shape presented for the mean numbers of trials shown in Table 9. It may be reasonably asked why we did not use a more conventional factorial design for the betting experiment, which would have obviated the need for eliminating participants that had carried out fewer than the fifty bets requested. However, we reasoned that there were at least two things that militated against the more conventional approach; first, the motivation of the participants was reasoned to be greater in a situation where it could be percieved that they had some control and could stop at any time, rather than being forced to complete a set number of trials; along with this, second, we wanted to keep dropouts to a minimum, particularly in view of the lower level of control we had with an internet based experiment (as it turned out we had an exclusion rate on numbers of bets of 27%, which would seem, intuitively, quite high, however, we currently do not have an objective comparison); and third, we considered our design a better facsimile of how people learn ‘in the real world’.

**Table 9:** Descriptive statistics for the presentations of shapes.

Shape	Mean presentations	SD
Star	18.74	7.29
Pentagon	17.41	8.89
Circle	17.67	8.19
Hexagon	19.59	6.72
Triangle	16.56	7.59
Square	18.59	8.13

### Factor Scores

Of the different reward distributions, there were two shapes where the probability of something happening was high and the probability of winning was low, two shapes where the probability of something happening was high and the probability of winning was high and two shapes where the probability of something happening was neither low nor high and the probability of winning was also neither low nor high. The result of these different distributions is that two shapes were ‘bad’, two shapes were ‘good’ and two shapes were indifferent.

**Figure 10:**
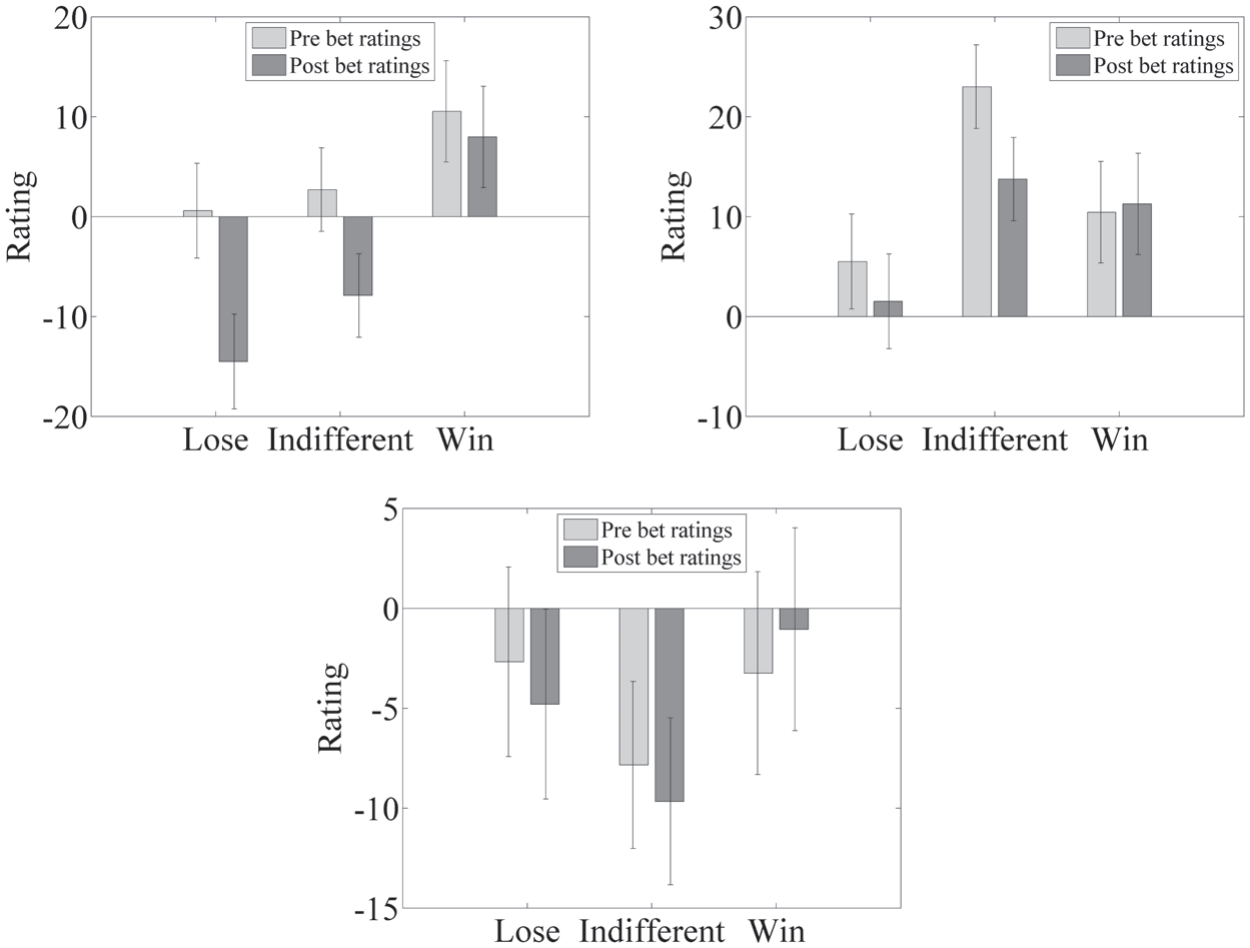
Ratings for the initial (light bars) and experimental (dark bars) semantic differentials. The left panel shows ratings for the Evaluation dimension for each combined distribution of shapes, the right panel ratings for the Potency dimension and the bottom panel the activity dimension. Ratings were between −50 to 50 with 0 indicating neutral. Error bars are standard error of the mean.

**Figure 11:**
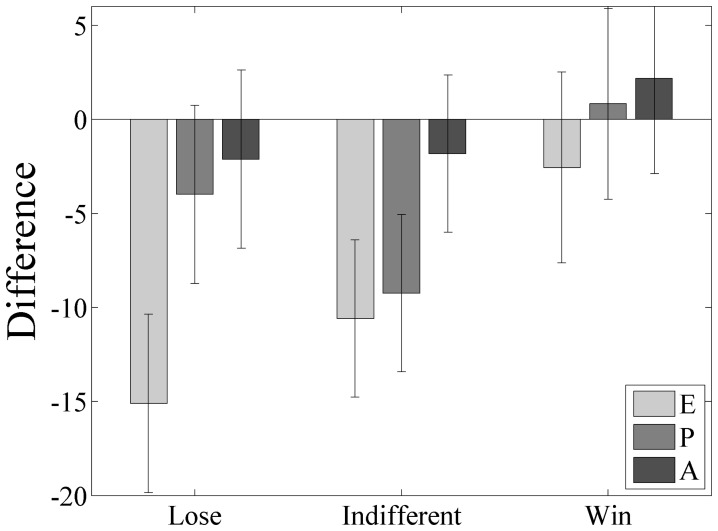
The differences between the initial and experimental semantic differential for Evaluation (lighter left bars), Potency (darker middle bars) and Activity (darkest right bars) for each combined shape distribution. Error bars are standard error of the mean.

Factor scores were calculated using the factor score coefficients established in the factor analysis for the initial ratings experiment (see Table 8). To make the results more comprehensible the reward distributions were reduced from six to three by combining the good, bad and indifferent distributions identified in Table 5; both semantic differentials are shown together in Figure 10, where the differences can be seen. However, perhaps more illustrative are just the differences shown for each factor of the semantic differential in Figure 11. ***Note that whether the pairwise differences between the combined shapes are or are not significant is not of interest here, only that the series of betting trials has influenced the semantic differential.***

### Reward Histories

The numbers of good (wins), bad (losses) and indifferent outcomes were also recorded for the experiment and these were collapsed across participants and shapes in order to provide a basis to investigate the relationship between the reward histories for each of the shapes and the semantic differential for the shapes. In order to do this the same measures of reward as for the blog search data discussed above, were calculated and correlated with Evaluation, Potency and Activity.

Although they were modest, significant correlations suggest that both Evaluation and Potency were changeable by being exposed to the different reward distributions in the shapes experiment. In particular, relative reward, 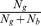, was correlated with the change in Evaluation, 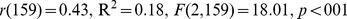; and negative reward, 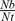, was correlated with Potency, 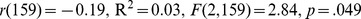. There was no significant correlation for Activity, 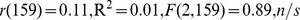. Taken together, these results suggest that, at least for the Evaluation and Potency dimensions, the semantic differential has been significantly influenced by the series of bets carried out by participants.

## Discussion

In this paper we started out with the observation that a broader representation of reward, rather than the single dimension usually assumed, is needed in order to account for choice making behaviour: To make effective choices in the face of uncertainty, we need to take into account not only reward but risk and uncertainty as well. Here we showed that a richer reward structure can be formed from good and bad experiences, and that Osgood's [1] semantic differential can be considered to represent this structure.

When factor analysis is applied to rating scales in a very large number of domains, three factors emerge: Evaluation, Potency, and Activity. While this has been known for over 50 years, why it happens has been less clear. Here we propose that these three factors are, in fact, a representation of the history of reward associated with a concept and that this representation of reward is required whenever we need to compare and choose between alternatives. What does this representation look like?

The factor that almost always captures the most amount of variance in the semantic differential is Evaluation. This we found was very strongly correlated with the proportion of rewarded events that were positively rewarded (technorati 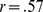, blogscope 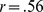): Evaluation is to first approximation simply relative reward. Perhaps the most surprising thing about this is that there is essentially no correlation with the absolute proportion of positively rewarded events (technorati 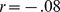, blogscope 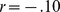): Evaluation does not measure the probability of good events happening, but the ratio of good to bad. The second characteristic associated with Evaluation is the (log) frequency of occurrence: Things that happen more often are preferred to those that are infrequent. This is simply the well known Mere Exposure effect and once the semantic differential is identified with reward, it is not surprising to find this: Things that are commonly encountered (and therefore well understood) are preferred to objects that are only rarely evaluated.

The second dimension, Potency, was most strongly related to the absolute probability of negative reward (technorati 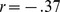, blogscope 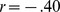). Potency essentially measures the risk (of bad things happening), making it clear why Potency needs to be represented for every object we can make decisions about: It is important not only to know the average reward associated with an option, but also how risky it is.

Our data analysis and experiments have less to say about the third dimension of the semantic differential, Activity, which was more modestly related to an interaction between risk and reward (technorati 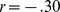, blogscope 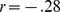). As well as a representation of the average relative reward and risk associated with an option, in order to make effective decisions, we also need to know how certain we are of this assessment. Uncertainty can come in two forms. Either we have limited and variable experience of a concept or, often more importantly, we have little control over the a thing or a situation. Concepts of high Activity (e.g. ones that are fast, noisy and active) are very often associated with less certainty and less controllability than ones that are slow, quiet and inactive. This is not directly measurable in either our blog analysis or shapes experiment, but to make effective decisions, we need to know the level of controllability/certainty associated with an option: we propose that Activity is that measure.

If the semantic differential represents our three dimensional reward structure as proposed and if the reward structure is based on good and bad experiences, then it will be possible to influence that reward structure and thereby the associated semantic differential through exposure to stimuli associated with varying rewards. Based on an approach reminiscent of the Iowa gambling task, which has been used extensively for investigating the Somatic Marker hypothesis [39], [40], the shapes experiment achieved this using arbitrary coloured shapes that were associated with differing reward distributions, in a betting game. It was hypothesised that providing participants with a sufficient number of random trials of these shapes should be enough to affect the reward summary for a given shape and be capable of changing the semantic differential associated with it.

An initial rating experiment, using the semantic differential technique, captured pre-existing connotative meaning for each of six arbitrary coloured shapes. The results of analysing the ratings provided by participants showed that, a semantic differential was formed with the expected dimensions of Evaluation, Potency and Activity. Although there is no discernible pattern across the shapes in the context of a simple rating experiment, participants were shown to have preconceived ideas about such simple things as coloured shapes.

A further semantic differential was calculated after a series of betting trials had taken place on the shapes, and found to be significantly different from the semantic differential produced from the initial ratings; this can only be attributable to the participants' reward structure and hence semantic differential, having been influenced by the reward statistics of the experimental shapes. Perhaps more interesting are the differences between the combined shape distributions for each of the semantic differential dimensions, shown in Figure 11.

Reward/Evaluation showed the greatest change between the initial and post betting ratings with the bad/lose shape combination attracting the largest difference and, as can be seen in Figure 11, being significantly different to the difference in reward for the good/win shape combination. Given that the participants were exposed to approximately the same numbers of trials for every shape, suggests that ‘more notice’ was taken of the more negative distributions, which might be considered to be consistent with loss aversion [41], where sensitivity to a loss is more acute than an equivalent win.

Sensitivity in the indifferent shapes combination included the risk/potency dimension, which showed a significant difference between pre and post betting. Considering this distribution of shapes as risk or danger is interesting because while these shapes had equal probability for a good or bad outcome, they also had equal probability of something happening or nothing happening. The result of this is that, although taken together these shapes are not as ‘objectively bad’ as those shapes in the losing distribution, they are somewhat tedious and considerably more difficult to predict, which is consistent with the argument that this constitutes greater risk and is presumably the reason for the worse ratings. Greater sensitivity to threats and potentially risky things seems to be evident even in the context of a simple betting experiment.

Identification of the semantic differential with reward allows us to speculate about the underlying neurophysiology. Reinforcement learning based models, particularly the 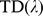 model [3], have had great success in developing an understanding of how we learn to make choices in an unknown world. The standard model uses a one dimensional representation of reward, associated with the action of dopamine [4], [42], [43]. This dopamine associated dimension clearly corresponds most closely to the Evaluation dimension of the semantic differential. The strong and significant relationship between the probability of a good experience in a rewarded situation, 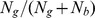, and Evaluation is particularly interesting because it accords very well with findings in the literature that dopamine neurons respond only to rewards, providing a reward prediction error that scales to the relevant range of magnitudes [5] maintaining the prior values represented in the prefrontal cortex. Accordingly, accurately predicted rewards and unrewarded events would be of no consequence and have no part to play in maintaining these values [4]. Though most computational models only have a single dimension of reward, more recent work has begun to look at *a*) punishment (or risk, corresponding to our Potency signal) and associated it with serotonin [17], [19], [44]; and *b*) uncertainty (Activity) and associated it with acetylcholine and norepinephrine [45], [46].

In conclusion, we propose that two representations from very different disciplines: reward (or utility), and the semantic differential, are in fact representations of the same thing. Identifying the semantic differential as a characterisation of reward offers a solution to the main theoretical issue with the semantic differential (what it is), and tells us why it is ubiquitous across domains, languages, and cultures. Almost all objects, actions and contexts need at some time to be compared with others (do I attend to this object or that, do I perform this action or another…). To do this we need to have an estimate of the reward associated with each alternative. We propose that the semantic differential is a representation of this, and “connotative meaning” is, to first approximation, a summary of reward history.

It also tells us potentially why three dimensions are needed: To make a choice, we need not only to know how rewarding an alternative is, but also how potentially dangerous it is and how sure we are of our prediction. A single dimension will be blind to risk and uncertainty, unable to efficiently balance exploration and exploitation, and choose options that, while of high average reward, could be associated with high levels of uncertainty and risk. The perils of making decisions that simply maximise reward, while ignoring risk and uncertainty, have been amply demonstrated by many of the transactions made before the credit crunch.
